# Dysphagia at 1 Year is Associated With Mean Dose to the Inferior Section of the Brain Stem

**DOI:** 10.1016/j.ijrobp.2023.06.004

**Published:** 2023-11-15

**Authors:** Eliana Vasquez Osorio, Azadeh Abravan, Andrew Green, Marcel van Herk, Lip Wai Lee, Deborah Ganderton, Andrew McPartlin

**Affiliations:** ⁎ Division of Cancer Sciences, University of Manchester, Manchester, United Kingdom; † European Molecular Biology Laboratory, European Bioinformatics Institute, Cambridge, United Kingdom; ‡ Departments of Clinical Oncology, Christie NHS Foundation Trust, Manchester, United Kingdom; § Speech and Language Therapy, Christie NHS Foundation Trust, Manchester, United Kingdom; ║ Radiation Medicine Program, Princess Margaret Cancer Centre, Toronto, Ontario, Canada; ¶ Department of Radiation Oncology, University of Toronto, Toronto, Ontario, Canada

## Abstract

**Purpose:**

Dysphagia is a common toxicity after head and neck (HN) radiation therapy that negatively affects quality of life. We explored the relationship between radiation therapy dose to normal HN structures and dysphagia 1 year after treatment using image-based datamining (IBDM), a voxel-based analysis technique.

**Methods and Materials:**

We used data from 104 patients with oropharyngeal cancer treated with definitive (chemo)radiation therapy. Swallow function was assessed pretreatment and 1 year posttreatment using 3 validated measures: MD Anderson Dysphagia Inventory (MDADI), performance status scale for normalcy of diet (PSS-HN), and water swallowing test (WST). For IBDM, we spatially normalized all patients’ planning dose matrices to 3 reference anatomies. Regions where the dose was associated with dysphagia measures at 1 year were found by performing voxel-wise statistics and permutation testing. Clinical factors, treatment variables, and pretreatment measures were used in multivariable analysis to predict each dysphagia measure at 1 year. Clinical baseline models were found using backward stepwise selection. Improvement in model discrimination after adding the mean dose to the identified region was quantified using the Akaike information criterion. We also compared the prediction performance of the identified region with a well-established association: mean doses to the pharyngeal constrictor muscles.

**Results:**

IBDM revealed highly significant associations between dose to distinct regions and the 3 outcomes. These regions overlapped around the inferior section of the brain stem. All clinical models were significantly improved by including mean dose to the overlap region (*P* ≤ .006). Including pharyngeal dosimetry significantly improved WST (*P* = .04) but not PSS-HN or MDADI (*P* ≥ .06).

**Conclusions:**

In this hypothesis-generating study, we found that mean dose to the inferior section of the brain stem is strongly associated with dysphagia 1 year posttreatment. The identified region includes the swallowing centers in the medulla oblongata, providing a possible mechanistic explanation. Further work including validation in an independent cohort is required.

## Introduction

Radiation therapy (RT) is an effective treatment that uses radiation to kill cancer cells.^1^ It is widely used and constitutes an important part of treatment in approximately 80% of patients with head and neck (HN) cancer, either as definitive or adjuvant treatment.^2^

Healthy tissues surrounding the tumor, including structures essential for swallowing, are unavoidably affected by RT.^3^, ^4^, ^5^, ^6^ Swallowing impairment (dysphagia) is a common side effect of HN treatment, and almost 50% of patients report it after RT.^2^, ^3^, ^4^, ^5^, ^6^, ^7^ Dysphagia following treatment is associated with higher risk of pneumonia, reduced oral intake, weight loss, malnutrition, prolonged tube feeding, and reduced quality of life.^5^ Gaining insights in normal tissue response and dysphagia would allow us to better design treatments, potentially allowing critical structures connected to dysphagia to be spared while maintaining tumor coverage.

Previous studies have identified associations between dysphagia and dose to structures throughout the head and neck, including the upper esophageal sphincter, larynx, pharyngeal constrictor muscles (PCMs), base of tongue, the floor of mouth and the genioglossus muscle, and salivary glands.^8^, ^9^, ^10^, ^11^, ^12^, ^13^ Reducing dose to some of these structures improves swallowing outcomes.^14^^,^^15^ However, the relative contribution of different structures to swallow dysfunction is unclear. As there is a large variety of organs, muscles, and glands reported, we aimed at exploring dose-effects relationship without a priori assuming which organs drive the effect. For this purpose, we used voxel-based analysis: image-based datamining (IBDM).

In this study, we used IBDM to explore the relation between RT dose to HN anatomy and dysphagia 1 year posttreatment, assessed using 3 different dysphagia measures.

## Methods and Materials

Sequential patients treated nonsurgically for oropharyngeal squamous cell carcinoma through a single HN cancer multidisciplinary team from June 2016 to May 2020 were included. Formal assessments of swallow function were performed by a speech and language therapist at baseline and at a follow-up appointment 1 year posttreatment. One hundred twenty patients were identified; 16 patients relapsed during follow-up and were excluded from analysis (Table 1). Clinical data and treatment data were collected from the ukCAT distributed learning database (ethics approval from the UK North West-Haydock Research Ethics Committee, reference number 17/NW/0060, local consent ref. 2021-018).

**Table 1:** Summary of data used in this study*

Variable	Statistic/value	Frequency
Patient characteristics		

T stage	1	10 (9.6%)
	2	47 (45.2%)
	3	18 (17.3%)
	4	29 (27.9%)
N stage	0	18 (17.3%)
	1+	86 (82.7%)
Performance status	0	72 (69.2%)
	1+	32 (30.8%)
Comorbidity score	0	62 (59.6%)
	1+	42 (40.4%)
HPV status (P16)	Positive	79 (75.9%)
	Negative	21 (20.2%)
	Missing	4 (3.9%)

Treatment characteristics		

Chemotherapy	No	27 (26.0%)
	Yes	77 (74.0%)
Dose prescription	55 Gy in 20 fx	3 (2.9%)
	66 Gy in 30 fx	98 (94.2%)
	70 Gy in 35 fx	3 (3.9%)

Treatment interventions		

Feeding tube insertion during treatment	No Yes	48 (46.2%) 56 (53.8%)
Feeding tube in situ at 1 y	No Yes	101 (97.1%) 3 (2.9%)

*Abbreviations*: fx = factions; HPV = human papillomavirus.

⁎ The authors’ institute follows a reactive protocol for feeding tube insertion during treatment.

### Dysphagia measurements

Three validated measurements of dysphagia were collected (Table 2).

**Table 2:** Dysphagia measures used in this study

Variable	Statistic/value	Frequency
Dysphagia measures at pretreatment		

WST	Pass Fail	83 (79.8%) 21 (20.2%)
PSS-HN	Mean, 87.5; SD, 21.2 Range, 30-100	
MDADI	Mean, 78.5; SD, 15.5 Range, 34.7-100	

Dysphagia measures at 1 y		

WST	Pass Fail	72 (69.2%) 32 (30.8%)
PSS-HN	Mean, 76.1; SD, 23.7 Range, 0-100	
MDADI	Mean, 75.6; SD, 17.1 Range, 21.5-100	

All patients had a measure before treatment and 1 year after treatment. PSS-HN and MDADI were treated as continuous variables.

*Abbreviations*: MDADI = MD Anderson Dysphagia Inventory; PSS-HN = performance status scale for normalcy of diet; SD = standard deviation; WST = water swallowing test.

#### Composite MD Anderson Dysphagia Inventory

The MD Anderson Dysphagia Inventory (MDADI) is a popular patient-reported outcome measure of swallowing-related quality of life. It is designed specifically for evaluating the impact of dysphagia on quality of life for patients with HN cancer. The MDADI has been validated regarding content, criterion, and construct validity and is considered reliable based on test-retest correlations.^16^ Widespread adoption of the MDADI in clinical research means that the MDADI is now arguably the principal patient-reported outcome tool used to measure dysphagia in HN cancer research.^17^ We used the composite MDADI score calculated using 19 items, excluding the global item.

#### Performance status scale for normalcy of diet

The performance status scale for normalcy of diet (PSS-HN) is a clinician-rated tool to assess interviews aimed at describing swallow performance. The PSS-HN has good interrater reliability and ability to discriminate levels of functioning.^18^ The normalcy of diet subscale, used in this study, is a scale from 0 to 100, with higher scores indicating better performance. This scale describes the type of food the patient is comfortable eating (eg, 50 indicates soft chewable foods, 80 all meats).

#### Water wallowing test

The 100-mL water swallowing test (WST) is a simple assessment which involves the patient swallowing 100 mL water as quickly and comfortably as possible.^19^ It is a validated and reliable swallowing measure for the HN cancer population and is sensitive for detecting patients with aspiration without x-ray or endoscopy. WST can be used as a timed, quantitative assessment. For this study, the WST was recorded as pass/fail. Patients failed the assessment if they coughed, had a wet voice quality postswallow, or were unable to complete the task.

As these tools measure varying aspects of dysphagia, they are correlated.^17^ For completeness’ sake, we also report their correlation in our data.

### Correlations between dysphagia measures and local RT dose

We applied IBDM, a voxel-wise analysis technique,^20^^,^^21^ to explore the associations between the local radiation dose and each of the measures collected 1 year after the end of treatment. This technique has 3 main steps: spatial normalization, region identification, and model construction. In the following sections, specific details for each step relevant to this study are given.

#### Spatial normalization

IBDM relies on mapping dose distributions of all patients to a reference anatomy using interpatient nonrigid registration. As previous studies reported dose to the swallowing muscles, to be correlated with dysphagia,^8^, ^9^, ^10^ we paid extra attention to the registration of the neck region. Additionally, to assess the impact of registration uncertainty, we used 3 different reference patients, all outside of the studied cohort.

For each reference patient, the same workflow was followed. We started by rigidly aligning the planning computed tomography scans for all patients to the reference anatomy, focusing on the base of the skull. Then, to align the neck region, we used the spinal cord delineations (available for all patients) to initialize a thin-plate spline,^22^ which was then used to create an intermediate floating image. Last, a full nonrigid registration (NiftyReg)^23^ was performed between the reference image and the intermediate neck-aligned image to find the final transformation. The resulting transformations were then applied to all patient's dose distributions to map them to the reference anatomies. As HN anatomy is assumed to be symmetrical, all patients were mirrored in the left-right axis and registered again to each reference patient to reduce any potential impact of target laterality.

We evaluated the quality of the registration both visually and quantitatively. For visual evaluation, we inspected the mean image intensity of all mapped images after registration (hereafter, mean registered image). For quantitative evaluation, we used 9 points of interest (POIs; Fig. E1) identified on an external data set of 100 HN planning computed tomography scans, which were registered to each reference patient using the same registration workflow. We calculated the standard deviation (SD) of the right-left (SD_RL_), anteroposterior (SD_AP_), and inferior-superior (SD_IS_) coordinates after mapping and reported them as a measure of interpatient registration consistency.

#### Identification of regions of significance

In the next IBDM step, voxel-wise statistics are performed to find regions where the dose is associated with the studied outcome.^20^ In this step, we used *t* test for the binary WST and Spearman correlation for the PSS-HN^24^ and MDADI scores, with all measures taken 1 year after the end of treatment. Notice that we treated both PSS-HN and MDADI as continuous variables in our analysis. Before voxel-wise analysis, we blurred all mapped dose distributions using a Gaussian convolution to account for registration uncertainty, with kernel defined by $\sigma_{RL}=SD_{RL},\;\sigma_{AP}=SD_{AP},$ and $\sigma_{IS}=SD_{IS},$ calculated after full nonrigid mapping. Additionally, we standardized all mapped dose distributions using the equivalent dose at 2 Gy per fraction (EQD2), assuming an α/β = 3 Gy for late effects, to account for different fractionations. Notice that we calculated the EQD2 doses locally using the fraction size delivered at each voxel. We use the unit Gy_EQD2_ for clarity.

As thousands of tests are performed simultaneously (1 test per voxel) for each outcome, we used permutation testing to correct for family-wise error rate.^20^^,^^25^ Family-wise error rate is the probability that at least 1 false discovery is made in an entire family of tests, where family refers to all the tests related to a particular experimental contrast^25^ (dose in each voxels vs outcome in this context). In permutation testing, the outcomes of the patients are randomly permuted, the voxel-wise statistics are performed again, and the extreme statistics (most negative, most positive) are recorded. Then, the *P* value of the extreme statistics in the nonpermuted configuration is estimated as the proportion of permutations that give a larger (or smaller) statistic. We used 1000 permutations and report the distribution of the extreme statistics. We explored regions consistently significant for all reference patients, with *P* ≤ .01. The overlap of all significant regions for the 3 regions for each reference anatomy was selected for the next step in the analysis.

#### Region evaluation

To evaluate the clinical significance of the identified region in the context of pretreatment factors and measures, we evaluated whether the planned dose to the identified region improved discrimination power in predictive multivariable models.

We first obtained the mean dose of the region of significance identified in the previous step for each reference anatomy. Notice we extracted the mean doses from the mapped dose distributions. We report reliability of the mean dose via intraclass correlation (ICC)^26^ and limits of agreement with the mean for multiple observers (LOAM).^27^ In our case, ICC assesses the consistency of the mean dose among the 3 reference patients. Similarly, LOAM determines the expected deviations of the mean dose among the 3 reference patients, and it is estimated as ±1.96 × the standard deviation of the differences. For the next part of the analysis, we used the averaged mean dose across the 3 reference anatomies.

Next, we found a clinical baseline model for each dysphagia measurement 1 year post-treatment by backward stepwise selection using all pretreatment variables listed in Table 1 (patient and treatment characteristics) and the corresponding pretreatment measurement listed in Table 2. We used multivariable logistic regression to predict WST and multivariable linear regression for PSS-HN and MDADI.

Improvement of model discrimination after adding the mean doses of the identified region was quantified using the Akaike information criterion at a significance level of .05. Statistical analysis was performed in R version 4.0.2.

#### Pharyngeal Constrictor Muscle dosimetry comparison

The relationship between mean dose to the PCM and dysphagia is well established^14^^,^^15^; therefore, discrimination improvement between models, including the mean dose of the identified region, was also compared with models including PCM dosimetry (namely mean dose to the superior-middle PCM [SMPCM] and inferior PCM [IPCM]). As we did not have SMPCM nor IPCM defined for any of our patients, we used a deep-learning auto-segmentation solution (Limbus AI, Regina, SK, Canada) to segment this structure in the 3 reference anatomies. We manually divided the resulting segmentation, which included all sections of the swallowing muscles in a single structure, to obtain SMPCM and IPCM according to guidelines.^28^ Then, we followed the same procedure described previously to collect the mean doses for the SMPCM and IPCM (including reliability assessment) and incorporated them in the model discrimination comparisons.

## Results

Patient and treatment characteristics are presented in Table 1, and their dysphagia scores are presented in Table 2. The correlation between all dysphagia measures is shown as a generalized pairs plot^29^ in Fig. E2.

### Spatial normalization

**Fig. 1:**
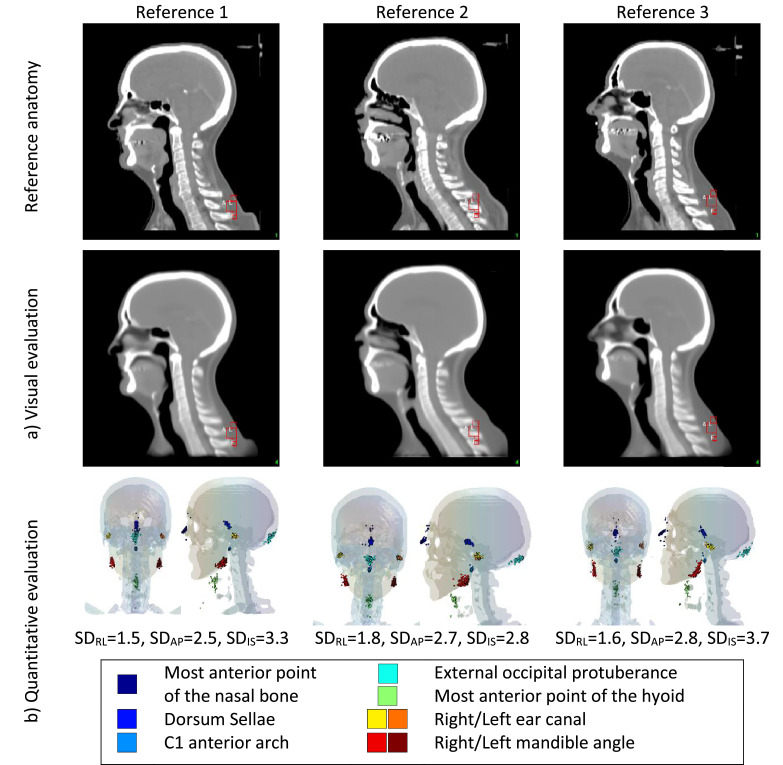
Evaluation of the registration workflow for each of the reference anatomies (top row). (a) Visual evaluation performed by visually inspecting the mean computed tomography after registration. (b) Quantitative evaluation computed the spread of 9 points of interest. For each reference anatomy, the frontal and lateral view of the patient's bony anatomy and the mapped points of interest are shown. Standard deviation (SD) of the right-left (SD_RL_), anteroposterior (SD_AP_), and inferior-superior (SD_IS_) coordinates (in mm) summarize the spread of the 9 mapped landmarks. See Figures E3 and E4 and Table E1 for additional details.

Figure 1 shows the evaluation of the registration workflow. Visual inspection of the mean registered image showed similar performance for the 3 reference patients with clear and sharp edges indicating limited residual discrepancies. See Fig. E3 for mean registered images after each workflow step. The quantitative evaluation shows a rather consistent registration, with SD of the identified POIs ranging from 1.5 to 1.8 mm in the RL direction, 2.5 to 2.8 mm in the AP direction, and 2.8 to 3.7 mm in the SI direction. See Fig. E4 and Table E1 for more details.

### Identification of regions of significance

**Fig. 2:**
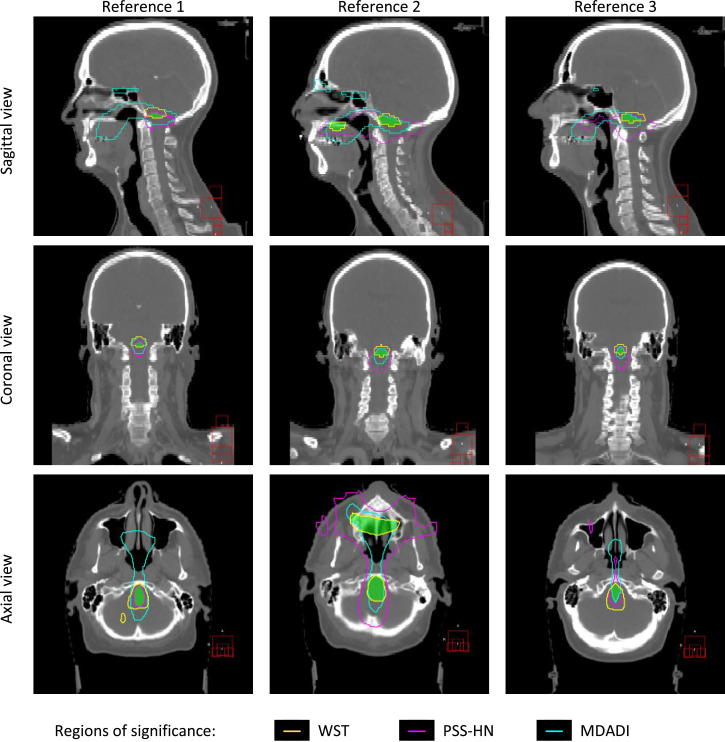
Regions of significance (*P* ≤ .005) shown for the 3 dysphagia measures and 3 reference patients. The green shadow indicates the overlap of the significant regions for the 3 different endpoints for each reference patient. *Abbreviations*: MDADI = MD Anderson Dysphagia Inventory; PSS-HN = performance status scale for normalcy of diet; WST = water swallowing test. (For interpretation of the references to colour in this figure legend, the reader is referred to the web version of this article.)

Permutation testing revealed significant association between dose and the 3 outcomes tested. Figure 2 shows the regions of significance for the 3 reference patients, with *P* = .005 for the WST region and *P* ≤ .0001 for PSS-HN and MDADI. High dose is significantly associated with WST failure 1 year after treatment. Conversely, high dose is associated with a lower score in PSS-HN and MDADI (Fig. E5). The dose to a region at the base of the brain stem was identified as highly associated for all outcomes. Additionally, a small region in the hard palate was identified in reference patient 2. To enforce consistency, we excluded this extra region for the rest of the analysis.

### Region evaluation and pharyngeal constrictor muscle dosimetry comparison

**Fig. 3:**
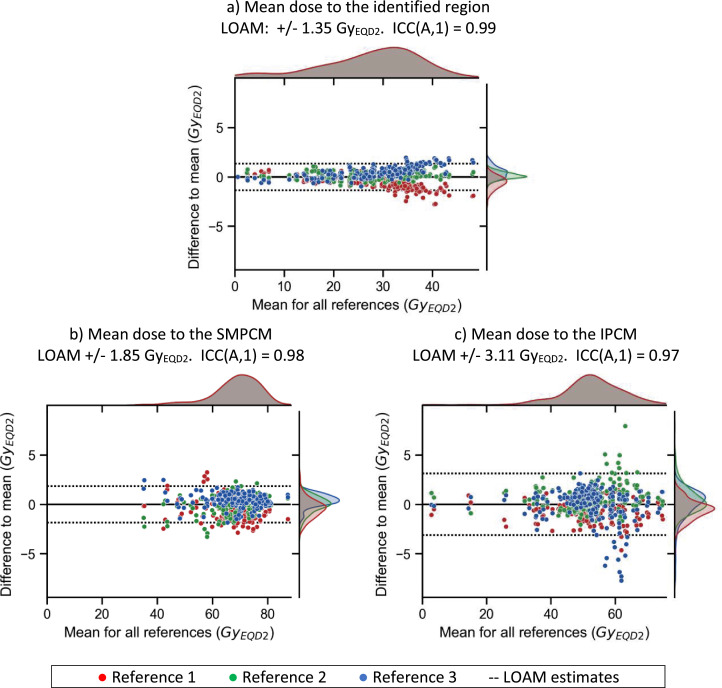
Agreement plots for mean doses of (a) the identified region, (b) superior-middle pharyngeal constrictor muscle (SMPCM), and (c) inferior PCM (IPCM). The scatter plot shows the mean dose collected from the mapped doses for each reference versus the difference to their mean, all in the equivalent dose at 2 Gy per fraction (Gy_EQD2_). The limits of agreement with the mean for multiple observers (LOAM) are shown in dashed lines. Additionally, the distribution of the mean and the differences for each reference are included in the top and right side of the scatter plot. *Abbreviation*: ICC = intraclass correlation.

Mean doses for the identified region ranged between 0.6 and 48.3 Gy_EQD2_ (Fig. 3a). Mean doses were reliable, with excellent consistency (ICC(A,1) = 0.99) and low deviations (LOAM, ±1.35 Gy_EQD2_). Mean doses for the SMPCM and IPCM ranged between 35.1 and 87.4 Gy_EQD2_ and 2.9 and 74.8 Gy_EQD2_, respectively, with excellent consistency (ICC ≥ 0.97) and low deviations (LOAM, ±1.85 Gy_EQD2_ for SMPCM and ±3.11 Gy_EQD2_ for IPCM; Fig. 3b, 3c). As all included patients have oropharyngeal tumors, parts of the SMPCM were often included in the high-dose clinical/planning target volume, explaining the large mean doses. Low correlation was found between the mean doses to the identified region and the SMPCM/IPCM (Kendall τ = 0.46 and 0.18, respectively).

Table 3 shows the improvement in discrimination for the models after including the mean dose to the identified region and PCM substructures. We found that all models including mean dose for the region significantly improved model discrimination (*P* ≤ .006). On the other hand, incorporating the mean doses to the PCM substructures resulted in models which improved discrimination but only significantly for WST (*P* = .04), borderline for PSS-HN (*P* = .06), and not significantly for MDADI (*P* = .26). When including the dosimetry of both the PCM substructures and the region, all models significantly improved discrimination (*P* ≤ .007). See Tables E2 to E4 for the complete model definition with the full set of multivariate variables and coefficients.

**Table 3:** Summary of models, including AIC and the *P* value associated with AIC improvement (with respect to the clinical baseline model) after including mean of the identified region and the constrictor muscle substructures (SMPCM_mean and IPCM_mean)

Model	AIC	*P* value
WST at 1 y (logistic regression)		
Variables selected: performance status, HPV status (P16), and pretreatment WST		

Clinical baseline model	205.5	Ref
+ region_mean	188.9	<.001
+ SMPCM_mean + IPCM_mean	199.3	.04
+ region_mean + SMPCM_mean + IPCM_mean	191.8	.001

PSS-HN at 1 y (linear regression)		
Variables selected: performance status, comorbidity score, and pretreatment PSS-HN		

Clinical baseline model	130.3	Ref
+ region_mean	115.7	<.001
+ SMPCM_mean + IPCM_mean	124.6	.06
+ region_mean + SMPCM_mean + IPCM_mean	113.6	<.001

MDADI at 1 y (linear regression)		
Variables selected: T stage, performance status, comorbidity score, and pretreatment MDADI		

Clinical baseline model	15.85	Ref
+ region_mean	5.45	.006
+ SMPCM_mean + IPCM_mean	13.15	.26
+ region_mean + SMPCM_mean + IPCM_mean	5.88	.007

*Abbreviations*: AIC = Akaike information criterion; HPV = human papillomavirus; IPCM_mean = mean dose to the inferior pharyngeal constrictor muscle; MDADI = MD Anderson Dysphagia Inventory; PSS-HN = performance status scale for normalcy of diet; Ref = reference; region_mean = mean of the identified region; SMPCM_mean = mean dose to the superior-middle pharyngeal constrictor muscle; WST = water swallowing test.

Smaller AIC values indicate better discrimination power for the models. For full model definition, see Tables E2 to E4.

## Discussion

We found that dose to the inferior section of the brain stem is strongly associated with dysphagia 1 year after HN RT. The identified region is in the medulla oblongata, where the swallowing centers responsible of coordinating swallowing motion are located.^6^^,^^30^ This provides a possible mechanistic explanation and insight in the decreased coordination between the various phases of the swallowing process observed in posttherapy swallowing studies.^31^^,^^32^ To the best of the authors’ knowledge, this association has not been reported before.

To evaluate the clinical significance of the mean dose to the identified region in the context of pretreatment clinical factors and measures, we quantified its contribution to improving prediction model discrimination. Our particular interest on pretreatment variables lies on the possible translation case, where the dose to the region could be used to personalize follow-up for patients and ultimately improve treatment planning. In this context, all models including mean dose to the identified region significantly improved discrimination (*P* ≤ .006). Moreover, we compared the prediction power of the dose to the identified region to the prediction power of a well-established relationship: mean dose to the PCMs and dysphagia.^14^^,^^15^ We found that mean dose to SMPCM and IPCM significantly improved discrimination for WST (the most objective measure), but not for PSS-HN nor MDADI. All models including both the mean dose to the region and the PCM substructures significantly improved discrimination. We hypothesize that different mechanisms responsible for radiation-induced dysphagia^3^^,^^9^ may be driven by dose-induced damage to different anatomic regions (which could the underlying reason for so many different structures being associated with dysphagia in different studies). For example, the physical act of swallowing could be impaired by (1) muscle damage (associated to dose received by the constrictor and tongue muscles),^12^, ^13^, ^14^, ^15^ (2) saliva production disruption (associated to dose to the salivary glands),^11^^,^^12^ and (3) lack of coordination between swallowing structures (our newly discovered association of dose to the medulla oblongata). Moreover, there is evidence showing that cerebrovascular accidents (ie, strokes) in the lower part of the brain stem are correlated with dysphagia,^33^^,^^34^ which further supports our hypothesis. Notice that as our patient cohort is small, this hypothesis needs to be validated in an independent patient cohort before deriving dose thresholds to optimize treatments. Furthermore, as we relied on mapped doses to estimate the mean doses to the identified region, as well as for the SMPCM and IPCM, part of the validation work requires running a similar analysis in the patient's coordinates with the delineated structures.

As the association between dysphagia and feeding tube insertion during treatment has been previously suggested,^24^^,^^35^ we explored the impact of this factor in model performance (Table E5). This may indicate that dose to the brain stem is also associated with acute dysphagia leading to weight loss and feeding tube insertion during treatment, consistent with previous findings.^36^

Understanding of the association of dose to brain stem and risk of subsequent swallow dysfunction is currently limited. Only one study has previously considered the dose to medulla and late dysphagia with lower cranial neuropathy.^37^ No correlation was reported in this small study of 12 symptomatic patients, which defined late dysphagia as a minimum of 4 years normal function posttreatment before developing swallow dysfunction. This suggests differing underlying pathologic processes to dysfunction seen at 1 year. Beyond dysphagia, dose to the medulla has been associated with acute fatigue^38^^,^^39^ and nausea/vomiting.^40^^,^^41^

Regarding the variables selected for the clinical baseline models, performance status (PS) was selected for all models. PS is a relatively subjective measure assessed by visual inspection by a clinical specialist. Recently, assessing sarcopenia has been proposed as a quantitative alternative to PS.^42^ Additionally, sarcopenia has been linked to dysphagia.^43^^,^^44^ Further work including sarcopenia estimation and reassessing the model performance could help sharpen the insight on the effect of the dose to the identified region.

Gaining insights in brain stem involvement in dysphagia would allow us to better design treatments, potentially allowing the medulla oblongata to be spared while maintaining tumor coverage. Our hypothesis-generating findings should be further validated in independent patient cohorts before dose thresholds can be established. This will not be straightforward given that there is no optimal threshold for the PSS-HN. For the MDADI scale, a threshold of 60 (as used in NRG-HN002)^45^ or a 10-point difference (regarded as clinically meaningful)^17^ could be used as a minimally acceptable score. In this context, external cohorts, prospective trials, and/or approaches such as rapid learning^46^ can be used to clinically validate our findings. Simultaneously, possible sparing can be established via planning studies in retrospective data sets.

### Methodological considerations

The findings of our study showcase the strong advantage of using IBDM to explore local associations between dose and outcome as it does not rely on assumptions on any anatomic region before analysis. We were expecting to find strong correlations in the swallowing muscles/salivary glands,^8^, ^9^, ^10^ but instead, we found a correlation in the base of the brain stem. This association would have been completely missed in other types of analysis.

The region was found by a comprehensive analysis of 3 measures, quantifying different aspects of dysphagia 1 year after treatment. We observed a relation between region size and how multifactorial the measure is. In one extreme, the smallest region was found for WST that directly assesses physiological aspects, a very objective and simple measure. Next, we have PSS-HN, which assesses the type of food patients are comfortable eating in day-to-day life. This measure can be influenced by habits and preferences of the patient. Last, the largest region was found for the composite MDADI, which assesses the impact of dysphagia in the quality of life of the patients. This measure is the most comprehensive, including the emotional, functional, and physical aspects. The nature of dysphagia measures also explains the reduction of significance for models including mean doses to the SMPCM and IPCM (Table 3). Based on this observation, we would recommend driving voxel-wise analysis using measures that objectively measure an outcome to avoid spurious correlation in the region identification step. In particular, for MDADI scores, we would suggest analyzing the physical subscale independently for studies looking into linking dose and outcome (data not available in our case).

In this work the level of significance between the dose and dysphagia measures was high. We used thresholds corresponding to *P* = .005 for WST region and *P* ≤ .0001 for PSS-HN and MDADI. Notice that small differences in these thresholds generate regions of different volume in the patients (Figs. 2 and E6). These threshold values are present in the tail of the statistic distributions (Fig. E5), and they heavily depend on the number of permutations used during permutation testing. If all possible permutations were assessed, these thresholds would be more consistent between reference patients but at an excessive computational expense. Alternatively, we could use a pooled analysis to select a common threshold between all references or advanced methods generalizing the pareto distribution.^47^ This strict choice of significance (*P* ≤ .005) may also explain why we did not see the correlations in other regions. Exploring lower levels of significance may highlight regions with well-established associations with swallowing outcomes, such as the PCMs, tongue muscles, or salivary glands.

We have demonstrated the methodological advantage of using multiple reference patients. In our analysis, the region at the base of the brain stem was consistently identified in all reference anatomies while a small region in the hard palate was identified in a single reference anatomy. This difference likely arises from registration uncertainties and the threshold used to define the region (discussed in the previous paragraph). This highlights the limitations of this type of methodologies when a single reference is used. Performing a sensibility analysis, such as k-fold validation, or alternative approaches to reduce the impact of outliers, such as bootstrapping, could be used to improve the step of region identification. Other approaches can be used, such as using clustering strategy as proposed by Acosta et al.^48^

Image registration is at the heart of IBDM, as it spatially maps the dose distribution of multiple patients to a reference anatomy. As we were expecting to find correlations in the swallowing muscles, we put extra effort aligning the neck region using an intermediate step based on thin-plate splines.^22^ A clear improvement was observed, both visualized in mean registered images (Fig. E3) and confirmed by a decrease in SD_AP_ after neck alignment (Fig. E4, Table E1). Registration uncertainties directly affect the region identification step before model construction. In this context, not finding the constrictor muscles could also be attributed to the limited thickness of the structure with respect to the registration uncertainty. We evaluated the registration workflow using both visual and quantitative measures. We note, however, that the quantitative evaluation of the registration uncertainty relied on manually identified landmarks, mostly on bony anatomy. These landmarks are affected by inherent anatomic differences between patients (eg, length of nasal bone or jaw angle) and observer variation and likely introduce extra uncertainties estimating registration performance. Regarding dose collection for model construction, registration uncertainties did not considerably affect the mean doses. We evaluated this using ICC coefficients and the LOAM (Fig. 3a). To the best of the author's knowledge, this is the first time that uncertainty for dose statistics for identified regions has been evaluated before model construction.

## Conclusion

In this hypothesis-generating study, we found that dose to the inferior section of the brain stem is strongly associated with dysphagia at 1 year after HN RT. The identified region, located in proximity to the medulla oblongata, was found by a comprehensive analysis of 3 validated dysphagia measures—WST, PSS-HN, and MDADI—and indicates a new organ at risk for swallowing function.

## Appendix Supplementary materials

Supplementary materials Image, application 1

## Footnotes

Supplementary material associated with this article can be found in the online version at doi:10.1016/j.ijrobp.2023.06.004.
