# Supplementary material for: Dysphagia at 1 Year is Associated With Mean Dose to the Inferior Section of the Brain Stem
Source: Int J Radiat Oncol Biol Phys. 2023 Nov 15;117(4):903–13. doi: 10.1016/j.ijrobp.2023.06.004 (PMC10581448; doi:10.1016/j.ijrobp.2023.06.004)
Supplement: Supplementary materials [file mmc1.pdf]

Supplementary materials

DYSPHAGIA AT 1 YEAR IS ASSOCIATED WITH MEAN DOSE TO THE INFERIOR SECTION OF THE BRAINSTEM

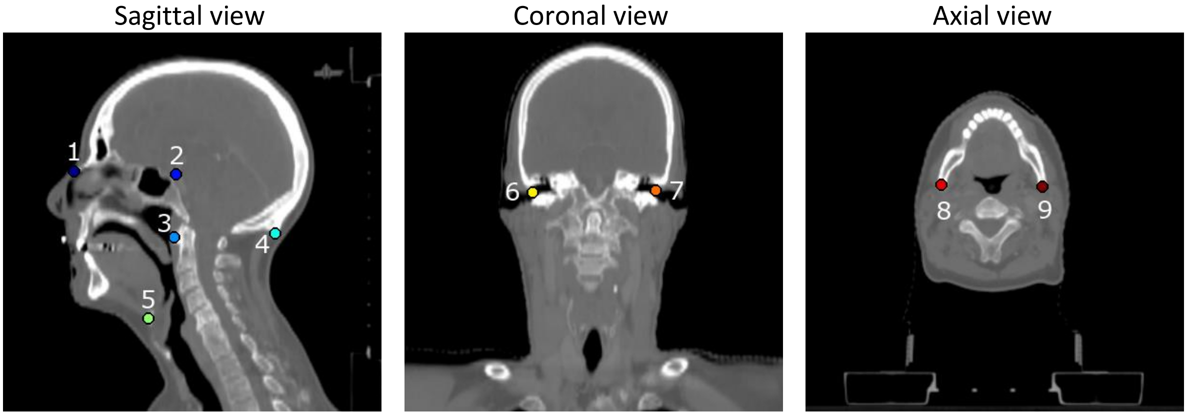

Figure S.1. Planning CT with the 9 points of interest identified for quantitative evaluation of the registration workflow. These are 1: Most anterior point of the nasal bone, 2: Dorsum Sellae, 3: C1 anterior arch, 4: External occipital protuberance, 5: Most anterior point of the hyoid, 6: Right ear canal, 7: Left ear canal, 8: Right mandible angle, 9: Left mandible angle.

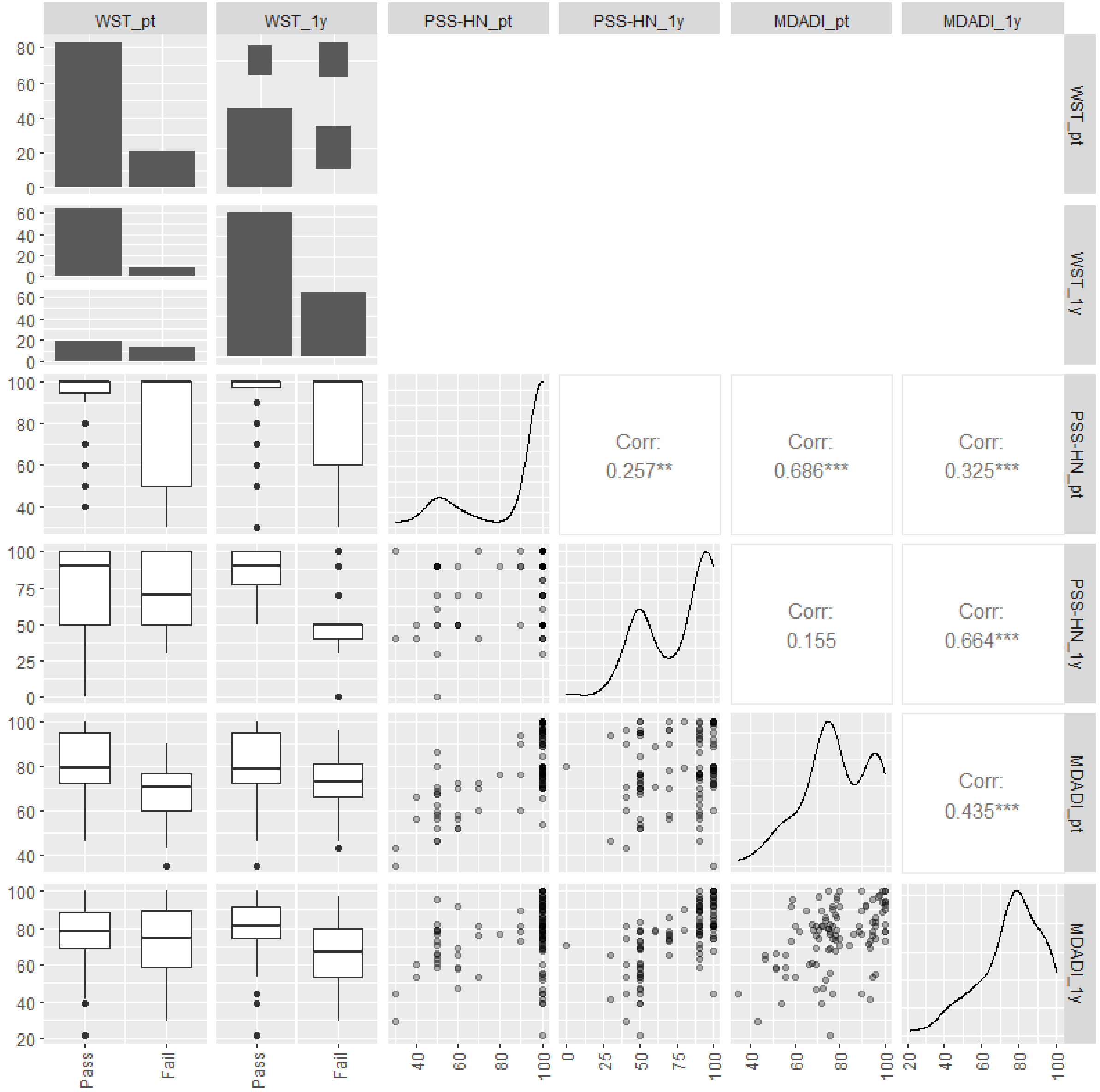

Prefix:
WST: Water swallowing test,
PSS-HN: Performance status for normalcy of diet
MDADI: M.D. Anderson Dysphagia Index

Postfix:
Measure taken at
_pt: pre-treatment
_1y: 1 year after treatment

Figure S.2. A generalised pairs plot showing the correlation between the dysphagia measures used in this study. Graphs in the diagonal show the histograms/density graph for each variable named at the top of the column/at the end of the row. Graphs below the diagonal show box-plot or scatter plots showing correlation between the variable named at the top of the column vs. the variable named at the end of the row. Conversely, for the continuous variables, the value shown above the diagonal denotes the Pearson correlation coefficient and its level of significance (** p<0.001, *** p<0.0001).

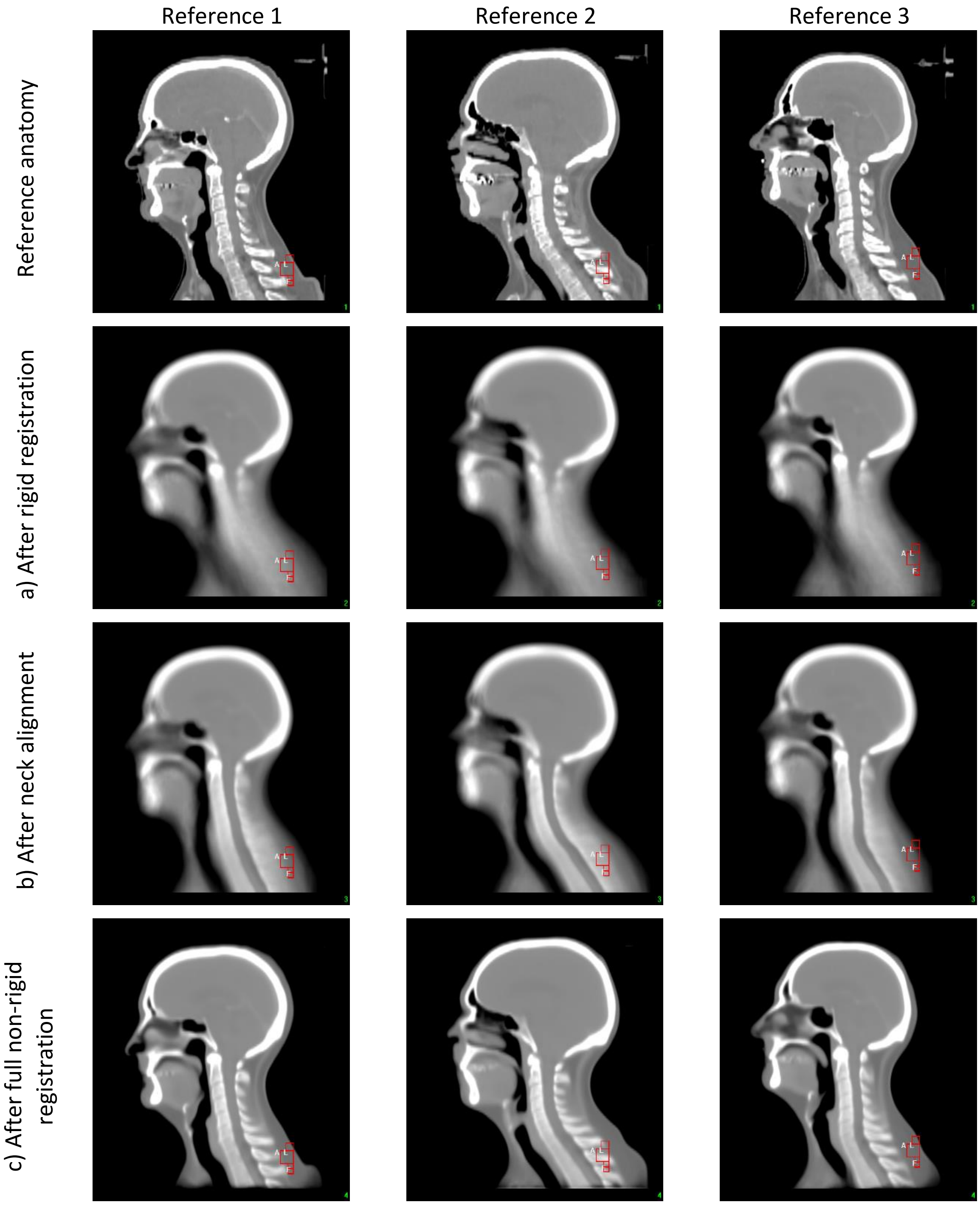

Figure S.3. Visual evaluation of the registration workflow for each of the reference anatomies (top row), detailing every registration step: mean registered images after a) rigid registration, b) neck alignment (thin-plate spline) and c) full non-rigid registration (NiftyReg). Notice the clear improvement in the neck region after the TPS neck alignment, and clear and sharp edges after full non-rigid registration.

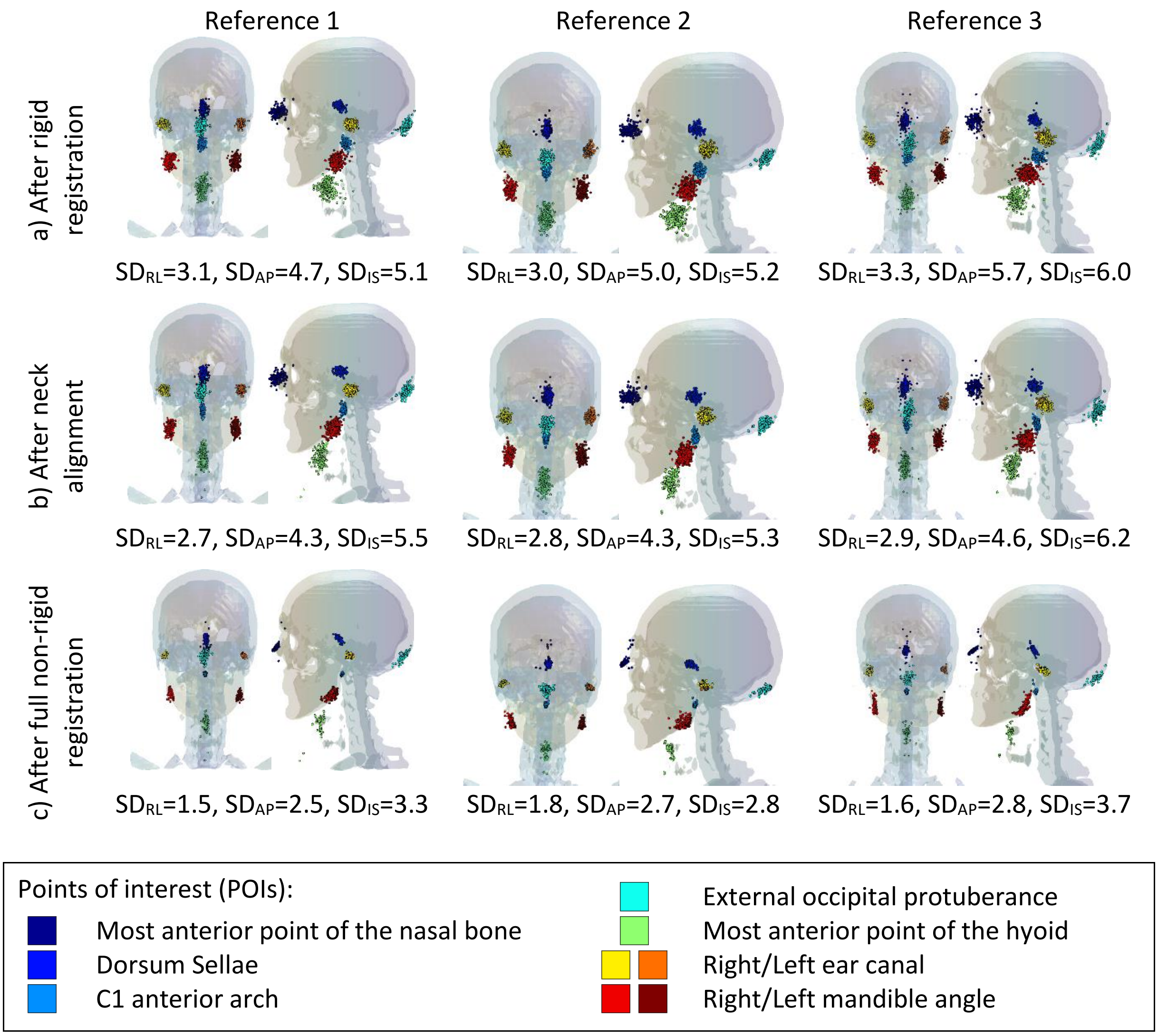

Figure S.4. Quantitative evaluation of the registration workflow for each of the reference anatomies measured after a) rigid registration, b) neck alignment (thin-plate spline) and c) full non-rigid registration (NiftyReg). In each cell, the frontal and lateral view of the reference patient's bony anatomy and the mapped POIs are shown. Below, the SD_{RL}, SD_{AP} and SD_{IS} in mm, summarise the spread of 9 mapped landmarks of the right-left (RL), antero-posterior (AP) and inferior-superior (IS) coordinates. See Table S.1 for extra details.

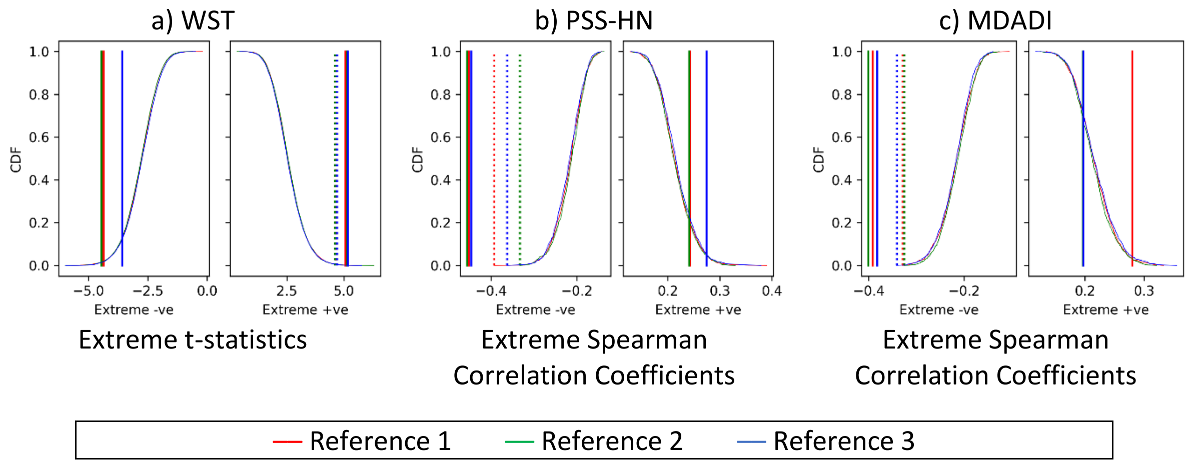

Figure S.5. Empirical cumulative distribution functions for the extreme statistics for each outcome resulting from permutation testing. For the negative statistics, the empirical cumulative distribution function (CDF) is shown for each reference patient. Conversely, the inverse CDF is shown for the positive statistics. Vertical solid lines indicate the extreme statistics for the non-permuted configurations. Higher doses are significantly associated with WST failure 1 year after treatment (positive t-statistics). Conversely, higher doses are associated with a lower score in PSS-HN and MDADI (negative Spearman correlation coefficients). We selected $p=0.005$ for identifying WST region and $p \leq 0.0001$ for PSS-HN and MDADI (dotted lines).

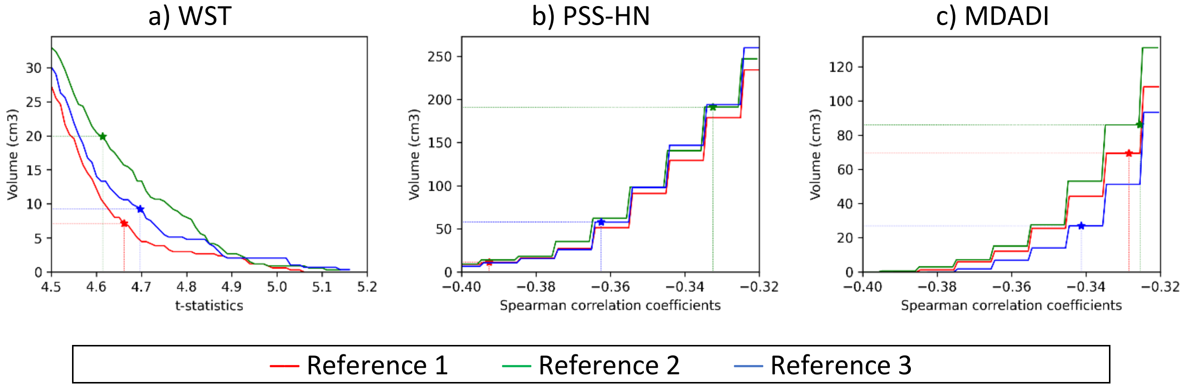

Figure S.5. Volume of regions corresponding to various statistics in close proximity to the selected threshold. The marker on the curve (*) indicates the selected statistic used in this study. Notice that the even though the curves follow similar trajectories, the volumes can change considerably between the selected thresholds (as they were selected independently). For example, the volume for reference 2 is approximately double the volume than that of references 1 and 3 for WST and more than triple for PSS-HN.

Table S.1. SD(POI) in mm for each POI and reference patient.

		Rigid registration			Neck Alignment			Full non-rigid registration		
		RL	AP	IS	RL	AP	IS	RL	AP	IS
Reference 1										
1	Nasal bone	2.9	5.9	5.6	2.9	5.9	5.7	1.2	2.1	4.1
2	Dorsum Sellae	1.4	3.7	3.5	2.0	4.2	3.5	1.3	2.6	3.0
3	C1 arch	2.4	3.0	3.9	1.3	1.8	4.1	0.7	0.7	1.1
4	Occipital Protuberance	3.6	4.8	5.4	3.1	5.0	5.4	2.7	4.1	4.3
5	Hyoid	4.1	8.3	9.4	3.1	5.7	11.7	1.7	3.4	7.4
6	Right Ear Canal	3.5	3.7	3.2	3.0	3.4	3.2	1.4	1.2	1.6
7	Left Ear Canal	2.4	3.1	3.0	2.6	3.4	3.0	1.3	1.1	1.3
8	Right Mandible Angle	3.9	5.0	6.0	3.2	4.9	6.3	1.6	3.3	3.5
9	Left Mandible Angle	3.4	4.6	6.1	3.0	4.6	6.4	1.7	3.6	3.0
	Average	3.1	4.7	5.1	2.7	4.3	5.5	1.5	2.5	3.3
Reference 2										
1	Nasal bone	3.1	6.6	5.5	3.1	6.3	5.5	1.9	2.7	4.6
2	Dorsum Sellae	1.3	4.2	3.9	2.2	4.3	3.9	1.5	2.6	2.3
3	C1 arch	2.2	3.0	4.2	1.5	1.8	4.4	0.8	0.8	1.6
4	Occipital Protuberance	3.5	4.8	4.5	3.2	5.2	4.5	3.1	4.8	2.5
5	Hyoid	4.1	8.1	9.1	3.2	4.7	9.7	2.0	1.8	4.9
6	Right Ear Canal	2.9	4.4	3.3	2.9	3.9	3.2	1.5	1.7	1.4
7	Left Ear Canal	2.7	3.5	3.7	2.8	3.5	3.7	1.6	1.8	1.3
8	Right Mandible Angle	3.6	5.5	6.4	3.1	4.9	6.3	2.0	4.0	3.7
9	Left Mandible Angle	3.6	5.0	6.6	3.2	4.4	6.5	1.4	4.1	2.8
	Average	3.0	5.0	5.2	2.8	4.3	5.3	1.8	2.7	2.8
Reference 3										
1	Nasal bone	3.3	5.8	7.9	3.3	5.7	7.9	1.5	3.0	4.7
2	Dorsum Sellae	1.4	3.6	4.7	2.2	4.6	4.8	1.4	2.2	3.5
3	C1 arch	2.6	4.6	4.4	1.4	2.0	4.6	0.7	0.9	2.2
4	Occipital Protuberance	4.2	5.4	7.2	3.5	4.9	7.0	3.2	4.2	3.4
5	Hyoid	4.6	9.4	9.4	3.5	5.8	10.5	1.9	2.2	5.2
6	Right Ear Canal	3.3	4.9	4.0	2.8	4.0	4.1	1.6	3.1	2.4
7	Left Ear Canal	2.6	3.9	4.1	2.8	3.8	4.0	1.5	1.8	1.5
8	Right Mandible Angle	4.3	7.2	6.4	3.3	5.7	6.7	1.5	3.6	5.9
9	Left Mandible Angle	3.6	6.7	6.3	3.2	5.1	6.4	1.4	3.9	4.6
	Average	3.3	5.7	6.0	2.9	4.6	6.2	1.6	2.8	3.7

Table S.2. Multivariable logistic regression models predicting for water swallowing test (WST) 1 year after treatment (Failure coded as 1). Next to the clinical baseline, models including dosimetrical information are presented. Model 1: clinical baseline model plus mean dose to the identified region. Model 2: clinical baseline model plus mean dose to the PCM substructures. Model 3: clinical baseline model plus mean dose to the identified region and PCM substructures

	Clinical baseline	Model 1	Model 2	Model 3
Variables	Odd ratio (p)	Odd ratio (p)	Odd ratio (p)	Odd ratio (p)
Intercept	0.15 (<0.001)	0.01 (<0.001)	0.00 (<0.001)	0.01 (0.008)
Performance Status				
1+ (Ref 0)	2.96 (0.003)	2.64 (0.012)	2.53 (0.014)	2.46 (0.022)
HPV status (P16)				
Negative (Ref positive)	2.87 (0.01)	2.68 (0.029)	2.90 (0.013)	2.77 (0.026)
Pre-treatment WST				
Fail (Ref pass)	4.88 (<0.001)	4.29 (0.001)	4.26 (0.001)	4.40 (0.001)
Mean dose to identified region (Gy_{EDQ2})	--	1.10 (<0.001)	--	1.10 (0.005)
Mean dose to SMPCM (Gy_{EQD2})	--	--	1.07 (0.029)	0.99 (0.821)
Mean dose to IPCM (Gy_{EQD2})	--	--	1.01 (0.747)	1.03 (0.356)
Model performance				
AUC	0.76	0.82	0.78	0.82
AIC	205.5	188.9	199.3	191.8
Delta AIC p	Ref	<0.001	0.04	0.001

Table S.3. Multivariable linear regression models predicting for performance status for normalcy of diet (PSS-HN) 1 year after treatment. As the outcome is skewed, a log transformation including a shift, was applied to ensure linear regression convergence. All estimates have been corrected. Next to the clinical baseline, models including dosimetrical information are presented. Model 1: clinical baseline model plus mean dose to the identified region. Model 2: clinical baseline model plus mean dose to the PCM substructures. Model 3: clinical baseline model plus mean dose to the identified region and PCM substructures.

	Clinical baseline	Model 1	Model 2	Model 3
Variables	Coef (p)	Coef (p)	Coef (p)	Coef (p)
Intercept	45.94 (<0.001)	73.09 (<0.001)	92.19 (<0.001)	72.28 (<0.001)
Performance Status				
1+ (Ref 0)	0.89 (0.049)	0.92 (0.157)	0.92 (0.152)	0.93 (0.186)
Comorbidity score				
1+ (Ref 0)	0.89 (0.032)	0.89 (0.03)	0.89 (0.033)	0.91 (0.069)
Pre-treatment PSS-HN	1.01 (<0.001)	1.00 (<0.001)	1.00 (<0.001)	1.00 (<0.001)
Mean dose to identified region (Gy_{EDQ2})	--	0.99 (<0.001)	--	0.99 (<0.001)
Mean dose to SMPCM (Gy_{EQD2})	--	--	1.00 (0.323)	1.01 (0.234)
Mean dose to IPCM (Gy_{EQD2})	--	--	1.00 (0.126)	0.99 (0.019)
Model performance				
Adjusted R^2	0.15	0.21	0.18	0.23
AIC	130.3	115.7	124.6	113.6
Delta AIC p	Ref	<0.001	0.06	<0.001

Table S.4. Multivariable linear regression models predicting for M.D. Anderson Dysphagia Index (MDADI) 1 year after treatment. As the outcome is skewed, a log transformation was applied to ensure linear regression convergence. All estimates have been corrected. Next to the clinical baseline, models including dosimetrical information are presented. Model 1: clinical baseline model plus mean dose to the identified region. Model 2: clinical baseline model plus mean dose to the PCM substructures. Model 3: clinical baseline model plus mean dose to the identified region and PCM substructures.

	Clinical baseline	Model 1	Model 2	Model 3
Variables	Odd ratio (p)	Odd ratio (p)	Odd ratio (p)	Odd ratio (p)
Intercept	47.40 (<0.001)	58.12 (<0.001)	69.26 (<0.001)	60.09 (<0.001)
T stage				
Advanced: 3+ (Ref early: 1,2)	0.89 (0.002)	0.93 (0.072)	0.93 (0.051)	0.95 (0.167)
Performance Status				
1+ (Ref 0)	1.08 (0.083)	1.10 (0.031)	1.10 (0.035)	1.10 (0.025)
Comorbidity score				
1+ (Ref 0)	0.91 (0.022)	0.91 (0.026)	0.91 (0.023)	0.92 (0.052)
Pre-treatment MDADI	1.01 (<0.001)	1.01 (<0.001)	1.01 (<0.001)	1.01 (<0.001)
Mean dose to identified region (Gy_{EDQ2})	--	0.99 (<0.001)	--	0.99 (0.003)
Mean dose to SMPCM (Gy_{EQD2})	--	--	1.00 (0.225)	1.00 (0.514)
Mean dose to IPCM (Gy_{EQD2})	--	--	1.00 (0.327)	1.00 (0.082)
Model performance				
Adjusted R^2	0.18	0.22	0.20	0.23
AIC	15.85	5.45	13.15	5.88
Delta AIC p	Ref	0.006	0.26	0.007

Table S.5. Model discrimination changes after including feeding tube insertion during treatment.

Model	Pre-treatment variables		+ feeding tube	
	AIC	p-val	AIC	p-val
WST at 1year (logistic regression)				
Variables selected: performance status, HPV status (P16), and pre-treatment WST.				
Clinical baseline model	205.5	Ref	188.6	Ref
+ region_mean	188.9	<0.001	178.8	0.007
+ SMPCM_mean + IPCM_mean	199.3	0.04	188.7	--
+ region_mean + SMPCM_mean + IPCM_mean	191.8	0.001	182.1	0.04
PSS-HN at 1 year (linear regression)				
Variables selected: performance status, comorbidity score, and pre-treatment PSS-HN.				
Clinical baseline model	130.3	Ref	105.9	Ref
+ region_mean	115.7	<0.001	98.5	0.02
+ SMPCM_mean + IPCM_mean	124.6	0.06	106.5	--
+ region_mean + SMPCM_mean + IPCM_mean	113.6	<0.001	99.0	0.03
MDADI at 1 year (linear regression)				
Variables selected: T stage, performance status, comorbidity score, and pre-treatment MDADI.				
Clinical baseline model	15.85	Ref	-2.2	Ref
+ region_mean	5.45	0.006	-7.2	0.08
+ SMPCM_mean + IPCM_mean	13.2	0.25	-0.5	--
+ region_mean + SMPCM_mean + IPCM_mean	5.88	0.007	-5.2	0.23
